# Bioxolography Using Diphenyliodonium Chloride and *N*‐Vinylpyrrolidone Enables Rapid High‐Resolution Volumetric 3D Printing of Spatially Encoded Living Matter

**DOI:** 10.1002/adma.202501052

**Published:** 2025-04-26

**Authors:** Alexis Wolfel, Castro Johnbosco, Annalise Anspach, Marieke Meteling, Jos Olijve, Niklas Felix König, Jeroen Leijten

**Affiliations:** ^1^ Leijten Lab BioEngineering Technologies TechMed Centre Faculty of Science and Technology University of Twente Enschede 7522NB The Netherlands; ^2^ Rousselot BV Port Arthurlaan 173 Ghent 9000 Belgium; ^3^ xolo GmbH Volmerstraße 9B 12489 Berlin Germany

**Keywords:** bioxolography, high‐resolution, spatial patterning, volumetric bioprinting

## Abstract

Light‐based volumetric bioprinting enables fabrication of cubic centimeter‐sized living materials with micrometer resolution in minutes. Xolography is a light sheet‐based volumetric printing technology that offers unprecedented volumetric generation rates and print resolutions for hard plastics. However, the limited solubility and reactivity of current dual‐color photoinitiators (DCPIs) in aqueous media have hindered their application for high‐resolution bioprinting of living matter. Here, we present a novel three‐component formulation that drastically improves photoreactivity and thereby enables high‐resolution, rapid, and cytocompatible Xolographic biofabrication of intricately architected yet mechanically robust living materials. To achieve this, various relevant additives are systematically explored, which revealed that diphenyliodonium chloride and *N*‐vinylpyrrolidone strongly enhance D‐mediated photoreactivity, as confirmed by dual‐color photo‐rheology. This enables Xolographic bioprinting of gelatin methacryloyl‐based bioresins, producing >1 cm^3^ constructs at ≈20 µm positive and 125 µm negative resolution within minutes. Multimaterial printing, molecular patterning, and grayscale‐mediated mechanical patterning are explored to programmably create intricate, biomimetic, and concentration‐controlled architectures. We demonstrate the Bioxolographic printing of various cell types, showing excellent cell viability, compatibility with long‐term culture, and ability for nascent protein deposition. These results position Bioxolography as a transformative platform for rapid, scalable, high‐resolution fabrication of functional living materials with encoded chemical and mechanical properties.

## Graphical abstract

Bioxolography, a novel volumetric 3D‐bioprinting technique, enables rapid and high‐resolution fabrication of >1 cm^3^ engineered living materials. A newly developed three‐component photoinitiator system significantly enhances the photoreactivity of gelatin methacryloyl‐based bioresins, allowing for precise xolographic bioprinting. This platform supports multimaterial printing, concentration‐controlled molecular patterning, and grayscale‐mediated mechanical modulation to create complex, biomimetic, and spatially controlled architectures.

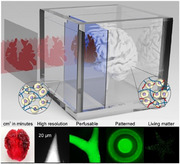

## 1 Introduction

Advances in biofabrication technologies hold the potential to engineer large‐scale (>1 cm^3^) tissues and organs that offer complex, multi‐scale, and multi‐functional architectures in clinically relevant timeframes.^[^ ^1^ ^]^ Current biofabrication approaches, however, face a significant trade‐off between speed and resolution. Techniques offering fine resolutions (e.g., two‐photon polymerization) typically require multiple days to fabricate cubic centimeter‐scale constructs,^[^ ^2^ ^]^ whereas faster techniques (e.g., extrusion‐based or lithographic printing) sacrifice resolution and normally only offer resolutions of hundreds of micrometers.^[^ ^3^ ^]^ Volumetric bioprinting technologies address these challenges by enabling rapid fabrication of sub‐centimeter constructs with resolutions in the tens of micrometers within minutes.^[^ ^4^ ^]^

The first reported volumetric bioprinting strategy utilized computed axial lithography, which operates by projecting 2D patterns onto a rotating vat of photopolymerizable bioresin, where spatial hardening occurs in regions where the light dose exceeds a polymerization threshold.^[^ ^1b^ ^]^ Although highly innovative, this technique has fundamental limitations that restrict resolution, reduce resin utilization efficiency, and hinder scalability due to sensitivity to vat size. Specifically, the threshold‐based approach relies on nonlinear resin properties to prevent premature polymerization, which demands complex algorithms and light feedback systems to mitigate issues such as reflections and shadowing during printing.^[^ ^5^ ^]^ These constraints inherently limit resolution, print depth, and resin utilization efficiency, as well as complicate user control over spatial patterning of anisotropic properties, such as stiffness^[^ ^6^ ^]^ and (bio)functionalization.^[^ ^7^ ^]^ Hence, novel volumetric bioprinting strategies to achieve rapid, high‐resolution biofabrication of the engineered living matter remain needed.

Xolography has been recently introduced as a novel volumetric 3D‐printing technology. Unlike computed axial lithography, Xolography employs a linear dual‐color photopolymerization mechanism, eliminating the need for nonlinear dose accumulation.^[^ ^8^ ^]^ Specifically, photopolymerization occurs by intersecting a UV‐light sheet with a visible‐light 4K projection within a movable print bath containing a photopolymerizable polymer and a dual‐color photoinitiator (DCPI). The DCPI transitions from a dormant spiropyran state to an active merocyanine form upon UV exposure (λ_1_), which is then excited by visible light (λ_2_) to initiate polymerization. This spatially confined crosslinking achieves resolutions at <10 µm within cubic centimeter‐scale constructs,^[^ ^9^ ^]^ surpassing computed axial lithography by order of magnitude without complex feedback systems, while also improving resin utilization efficiency and scalability.^[^ ^8^ ^]^ This has been associated with various innovations including Xolographic printing in flow,^[^ ^9^ ^]^ Xolographic printing of ceramics,^[^ ^10^ ^]^ and Xolographic printing in microgravity.^[^ ^11^ ^]^ However, reliance on DCPIs that suffer from poor water solubility and reactivity has challenged Xolography's ability to print hydrogels. Consequently, the potential of Xolography for fabricating hydrophilic (bio)materials and living matter has remained unexplored.

This study presents a novel bio‐ink formulation that enables high‐resolution Xolographic 3D‐printing of hydrogels and living constructs. To achieve this, we comprehensively explored various comonomers to enhance photopolymerization kinetics^[^ ^12^ ^]^ as well as introduced diphenyliodonium salt (DPI) as a tertiary photoinitiation component to boost initiation efficiency. This novel approach enables cytocompatible and effective Xolographic 3D‐printing of various hydrogel compositions including gelatin‐methacryloyl (GelMA), achieving micrometer‐scale resolution within minutes. Additionally, we demonstrate the versatility of this platform by fabricating multi‐material constructs and showcasing Xolography's unique capability for volumetric light‐dose patterning. Collectively, this platform enables the production of constructs with spatially encoded mechanical properties and molecular functionalization, which are critical for mimicking the architectural complexity of native tissues. These findings have significant implications for soft‐materials applications, especially for tissue engineering and regenerative medicine, advancing the development of volumetric bioprinting toward rapid, complex, and scalable engineering of living tissues for clinical applications.

## 2 Results and Discussion

### 2.1 Dual‐Color Photoinitiator for Aqueous (Bio)Inks

**Figure 1:**
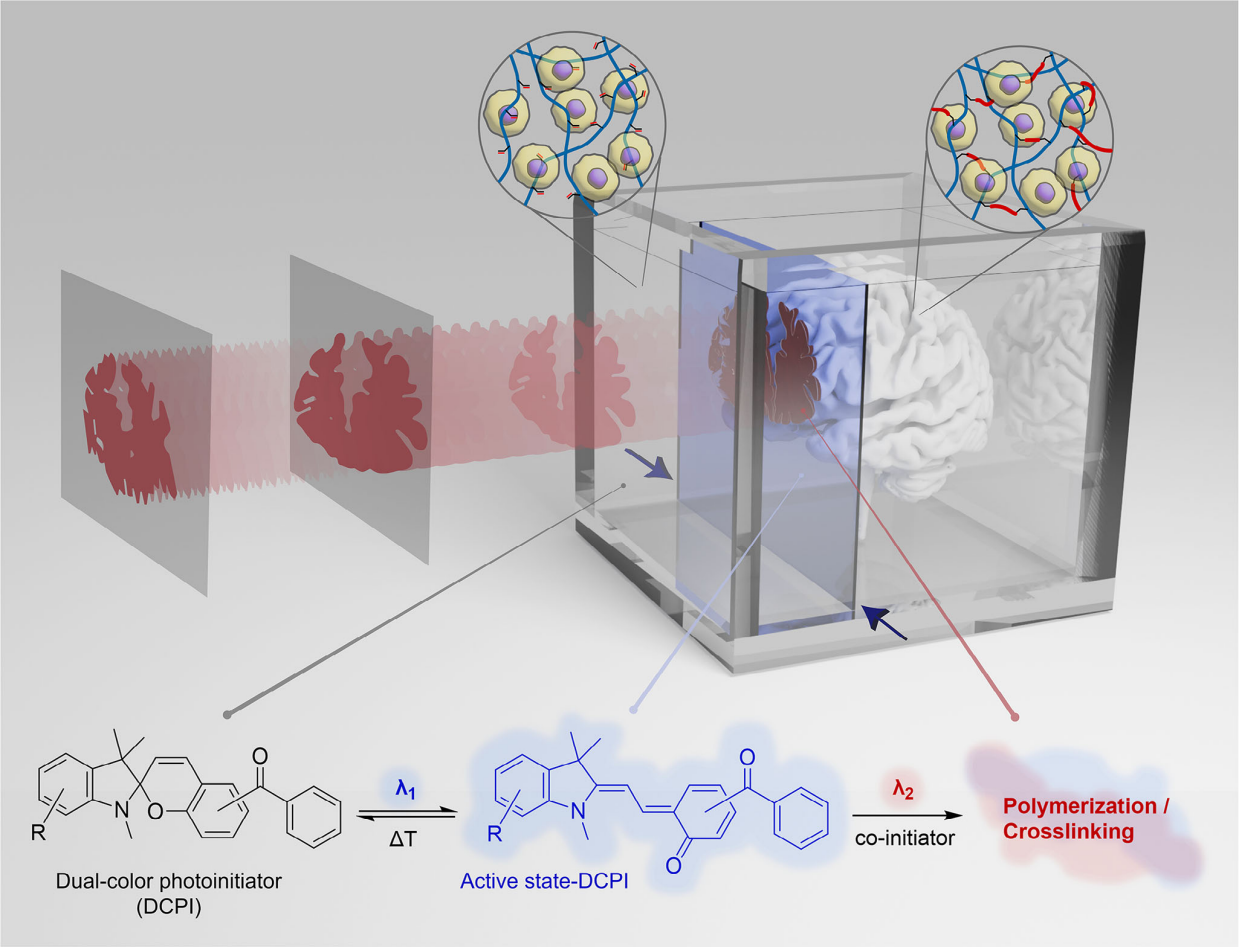
Schematic depiction of volumetric Bioxolographic printing. A transparent print bath containing cell‐laden photopolymerizable biomaterial solution and a DCPI undergoes voxelated crosslinking upon simultaneous exposure to two distinct light beams (λ_1_ + λ_2_). A UV light sheet (λ_1_) creates a thin photoreactive canvas by forcing the DCPI into its active state. Simultaneously, a video containing 2D slices of the printing object is projected (λ_2_) onto the photoreactive canvas, effectuating local polymerization where the two light beams intersect. A motorized stage moves the printing bath smoothly and continually away from the projector while the video is projected, thus fabricating complex yet seamless 3D living objects in high resolution within minutes.

**Figure 2:**
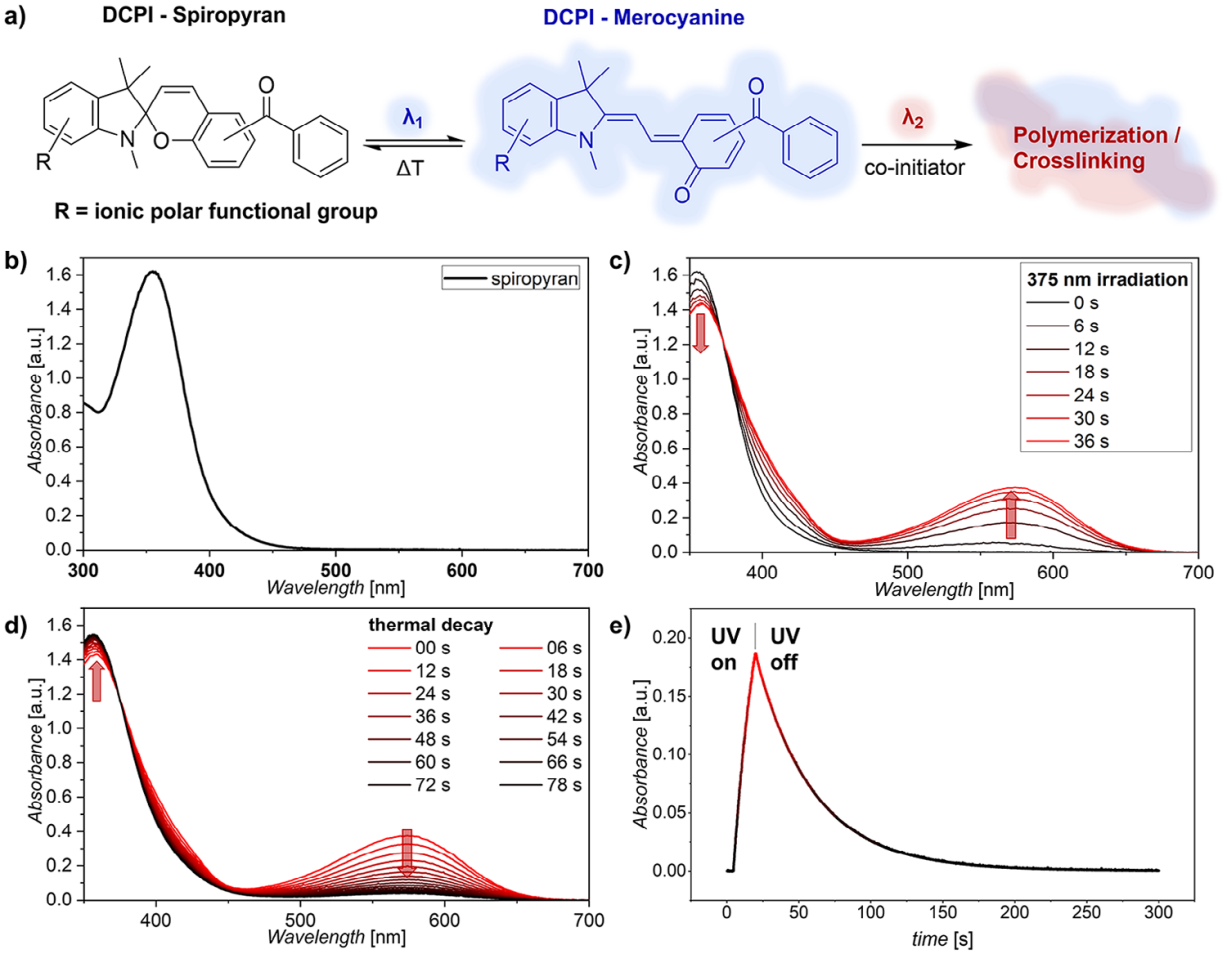
DCPI photochemical characterization. a) DCPI structure and its isomerization upon light irradiation. b) Absorbance spectrum of DCPI under dark conditions (spiropyran) shows c) an increase in merocyanine concentration under 375 nm UV irradiation; and d) a thermal relaxation under dark conditions following UV irradiation. Red arrows indicate the absorbance change of spiropyran/merocyanine species in time. e) Photoswitching kinetics followed at 570 nm, absorbance showing generation of the merocyanine state during 375 nm UV irradiation, followed by thermal relaxation in the dark.

Xolography allows for rapid and high‐precision 3D fabrication by orthogonally intersecting UV‐light sheets with a visible‐light 4K projection within a print bath of a photopolymerizable polymer containing a DCP (**Figure** **1**). The physicochemical properties and photoreactivity of DCPIs are thus of fundamental importance in realizing Xolographic printing. Recently, Xolography was introduced as a method to volumetrically print hydrophobic plastic resins, which relied on non‐water soluble DCPIs that are unsuited to print hydrogels.^[^ ^8^ ^]^ To address this challenge, we herein introduce a DCPI that was modified with an ionic functional group to enhance its solubility in aqueous environments (**Figure** **2**a). To evaluate its potential for hydrogel (bio)printing applications, we confirmed its photochemical properties in aqueous systems. Here, the DCPI's dormant state (i.e., spiropyran) exhibits UV‐light absorbance with a peak at λ_max_ 355 nm (Figure 2b). Upon irradiation at 375 nm, the spiropyran isomerizes to the merocyanine form (i.e., active state), which absorbs visible light with a peak at λ_max_ 575 nm (Figure 2c).

For preventing unintentional photocrosslinking in already addressed regions, behind the UV‐light sheet, it is essential for the DCPI to show a rapid deactivation. Hence, we investigated the relaxation behavior of the DCPI. Following interruption of the UV excitation, the merocyanine relaxed back to the spiropyran form with a half‐life of *t*_1/2_ of 28.0 s at 10 °C (Figure 2d,e). As temperature was expected to influence DCPI relaxation behavior, the effect of temperature was also evaluated. At room temperature (25 °C), the *t*_1/2_ decreased to 3.6 s, which is consistent with a reaction rate for the thermal relaxation process following the Arrhenius equation (Figure S1a, Supporting Information). This demonstrated that the DCPI exhibits a rapid relaxation at relevant temperatures for hydrogel bioprinting. As such, we assessed the DCPI's compatibility with hydrogel printing, by evaluating the DCPI's relaxation profile in a 10 wt% GelMA hydrogel, which was endotoxin purified to avoid any adverse cellular effects. This revealed that in GelMA, the *t*_1/2_ at 25 °C slightly increased to 4.1 s, which is likely due to the reduced molecular mobility and increased hydrophobicity of GelMA (Figure S1b, Supporting Information).^[^ ^13^ ^]^ Importantly, this relaxation rate is within the suitable range to facilitate Xolographic printing.^[^ ^8^ ^]^ These findings, therefore, underscore the potential of this water‐soluble DCPI to enable the Xolographic printing of hydrogels.

### 2.2 Xolographic 3D‐Printing of Biomaterials

**Figure 3:**
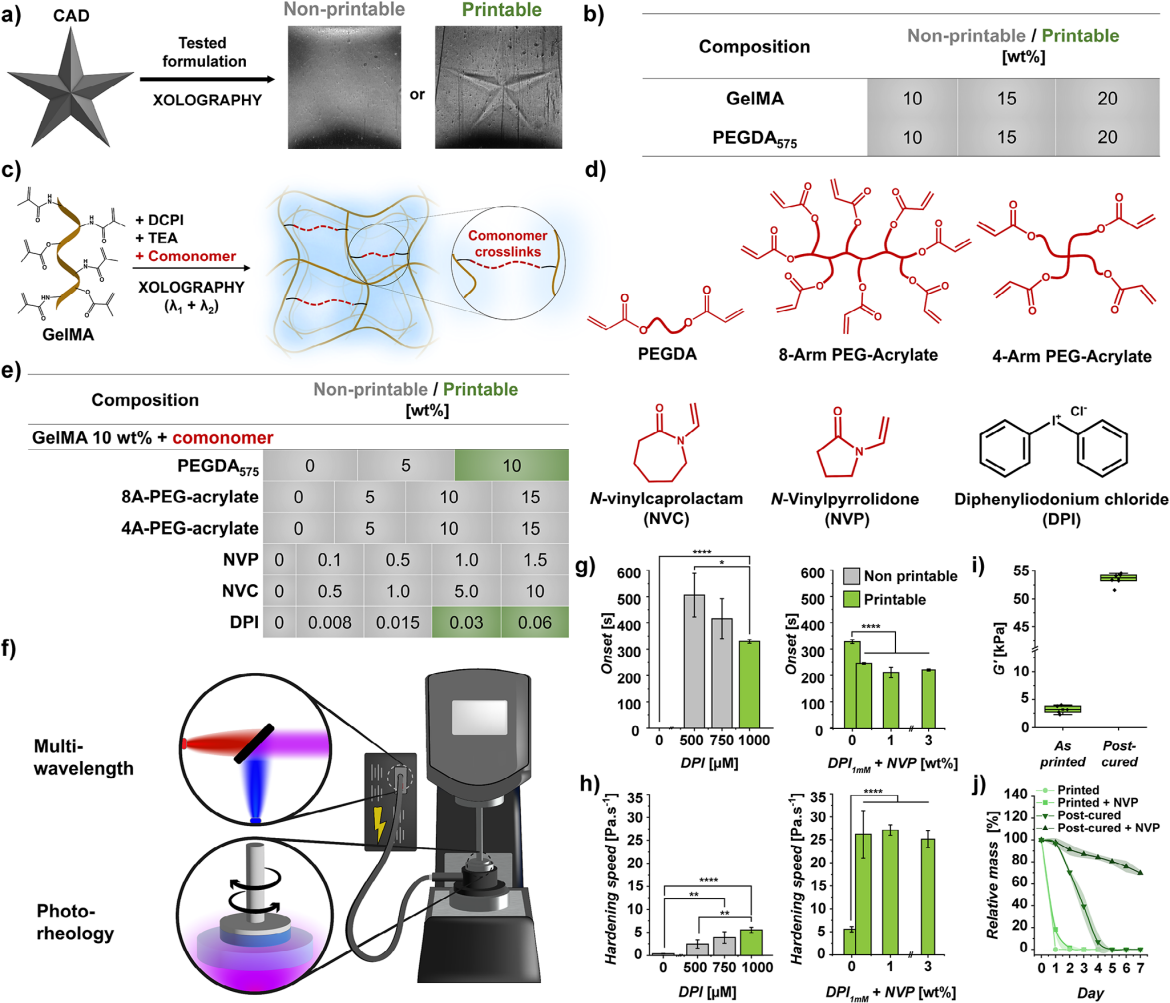
Bioresin formulation and reactivity optimization for the Xolographic printing of hydrogels. a) Xolographic 3D printing test and post‐printing characterization to evaluate printability of different formulations. b) Printability of tested GelMA and PEGDA‐based compositions (grey: non‐printable; green: printable). c) Schematic depicting the role of added comonomers to enhance photo‐crosslinking of (bio)inks. d) Comonomers (red) and DPI (black), which were tested on their e) effect on the printability of GelMA‐based bioresins (grey: non‐printable; green: printable). f) Schematic of the multi‐wavelength photorheology setup that was built to mimic the dual‐color photoinitiation mechanism of Xolography and investigate the curing kinetics of different bioresins. g) Effect of DPI and DPI+NVP on the photo‐crosslinking onset time and h) hardening speed of GelMA formulations. i) Storage modulus (G′) of Xolographically printed GelMA‐DPI‐NVP hydrogels immediately after printing and after post‐printing curing with UV+Vis light (375 nm + 565 nm) (*n* = 3). j) Collagenase‐IV‐mediated degradation of printed and post‐cured samples (*n* = 4).

We subsequently evaluated various hydrogel formulations to determine their suitability as bioresins for Xolographic printing. The assessment involved fabricating five‐point stars,^[^ ^14^ ^]^ with their physical presence analyzed using Schlieren imaging (**Figure** **3**a). Specifically, we confirmed that conventional GelMA and polyethylene glycol diacrylate (PEGDA) formulations could not be printed for any tested concentration (10‐20 wt%), even when subjected to high UV irradiation energies (>40 mJ mm^−2^) (Figure 3b). This confirmed that current DCPI‐based formulations possessed insufficient reactivity or conversion efficiency to allow for Xolographic printing of hydrogels. To address this challenge, we systematically explored the reactivity of GelMA‐based bioresins using a small library of comonomers and additives designed to improve photopolymerization efficiency (Figure 3c,d). Specifically, we investigated the use of low molecular weight monomers such as multi‐arm‐PEG‐acrylates,^[^ ^15^ ^]^ N‐vinylcaprolactam (NVC),^[^ ^12^, ^16^ ^]^ and N‐vinylpyrrolidone (NVP)^[^ ^12b^ ^]^ due to their reported ability to enhance the reactivity of GelMA resins in free radical polymerization processes for conventional light‐based, photo‐initiated cell‐encapsulation. Interestingly, adding 10 wt.% low molecular weight PEGDA (Mn 575) to GelMA allowed for successful Xolographic printing of hydrogels by enhancing the hardening speed and decreasing onset time during photopolymerization (Figure S2, Supporting Information). However, the use of low molecular weight PEG‐acrylates is associated with a high level of cytotoxicity,^[^ ^17^ ^]^ as confirmed via live/dead stain of Xolographically printed 3T3 fibroblastic cells (Figure S2, Supporting Information). We hypothesized that multi‐arm PEG‐acrylates could serve as better alternatives, given their lower cytotoxicity and frequent use in cell encapsulation.^[^ ^15^ ^]^ However, unlike low Mw PEGDA, 4‐arm, and 8‐arm PEG‐acrylates did not support xolographic printing. Additionally, we tested NVC and NVP, within reported cytocompatible concentration ranges, as both have been reported to accelerate GelMA crosslinking rate by forming short polymeric chains between methacryloyl residues.^[^ ^12^, ^16^, ^18^ ^]^ However, all tested concentrations failed to yield a Xolographically printable formulation, highlighting that conventional approaches to boost reactivity are insufficient to achieve Xolographic printing of hydrogels (Figure 3e). Therefore, a novel solution is needed to overcome the low photoinitiation efficiency of DCPIs and thus enable Xolographic biofabrication of living matter.

We pioneered the innovative design of a three‐component photoinitiation system to boost the photoreactivity during Xolographic printing. Thus far, Xolography has only employed a conventional Type II photoinitiation mechanism for Xolographic printing. DCPIs are used as photosensitizers in combination with a co‐initiator, usually triethanolamine (TEA), which acts as a reducing agent to form radicals through a conventional photoredox pathway.^[^ ^19^ ^]^ Innovatively, we herein upgraded the photoinitiation system with the oxidation agent diphenyl iodonium chloride (DPI), forming a three‐component system that enabled the Xolographic printing of engineered living materials (Figure 3e). To quantify the performance of the new photoinitiation system, we developed a dual‐color photorheology setup, which revealed that, even at low concentrations (1 mM), DPI significantly enhanced the reactivity of the ink (Figure 3f). Specifically, incorporating DPI reduced the polymerization onset time and accelerated the hardening rate (Figure 3g,h). It is reasoned that DPI achieves this by re‐converting DCPI free radicals (DCPI•) to their initial state (DCPI) through a photooxidative process while forming DPI‐based reactive radicals.^[^ ^20^ ^]^ This dual action could, therefore, slow down the consumption of DCPI during printing while additional DPI‐generated radicals speed up the free radical polymerization (Figure S3, Supporting Information).

We next investigated whether the addition of comonomers could further enhance DPI‐mediated photoreactivity, which revealed that NVP further accelerated Xolographic photopolymerization of GelMA hydrogels. Notably, the addition of even low concentrations of NVP (e.g., 0.3 wt.%) significantly boosted the hardening speed (4.7‐fold) and reduced the onset time (from 330 ± 7 s to 247 ± 3 s) (Figure 3g–h. Figure S4, Supporting Information). Xolographically printed GelMA‐DPI‐NVP hydrogels were characterized by a storage modulus of 3.2 ± 0.7 kPa, which allowed for robust handling during downstream use as well as providing a mechanical environment suitable for cell culture. On‐demand post‐crosslinking of Xolographically printed GelMA hydrogels allowed for attaining storage moduli as high as 54.2 kPa (Figure 3i), which therefore provides elasticity values suitable for applications such as soft robotics^[^ ^21^ ^]^ or wearable sensors.^[^ ^22^ ^]^ Remarkably, incorporating NVP improved the stability of printed constructs against collagenase IV‐mediated enzymatic degradation, likely by increasing the crosslinking degree and, consequently, their mechanical strength (Figure S4, Supporting Information). Additionally, applying a post‐curing treatment allows for further control over the mechanical properties and enzyme‐mediated degradation rate of the constructs. These findings underscore that small amounts of DPI and NVP synergistically enable Xolographic printing of aqueous polymer solutions for the rapid volumetric biofabrication of hydrogel constructs.

### 2.3 High‐Resolution Xolographic 3D‐Printing of Hydrogels

**Figure 4:**
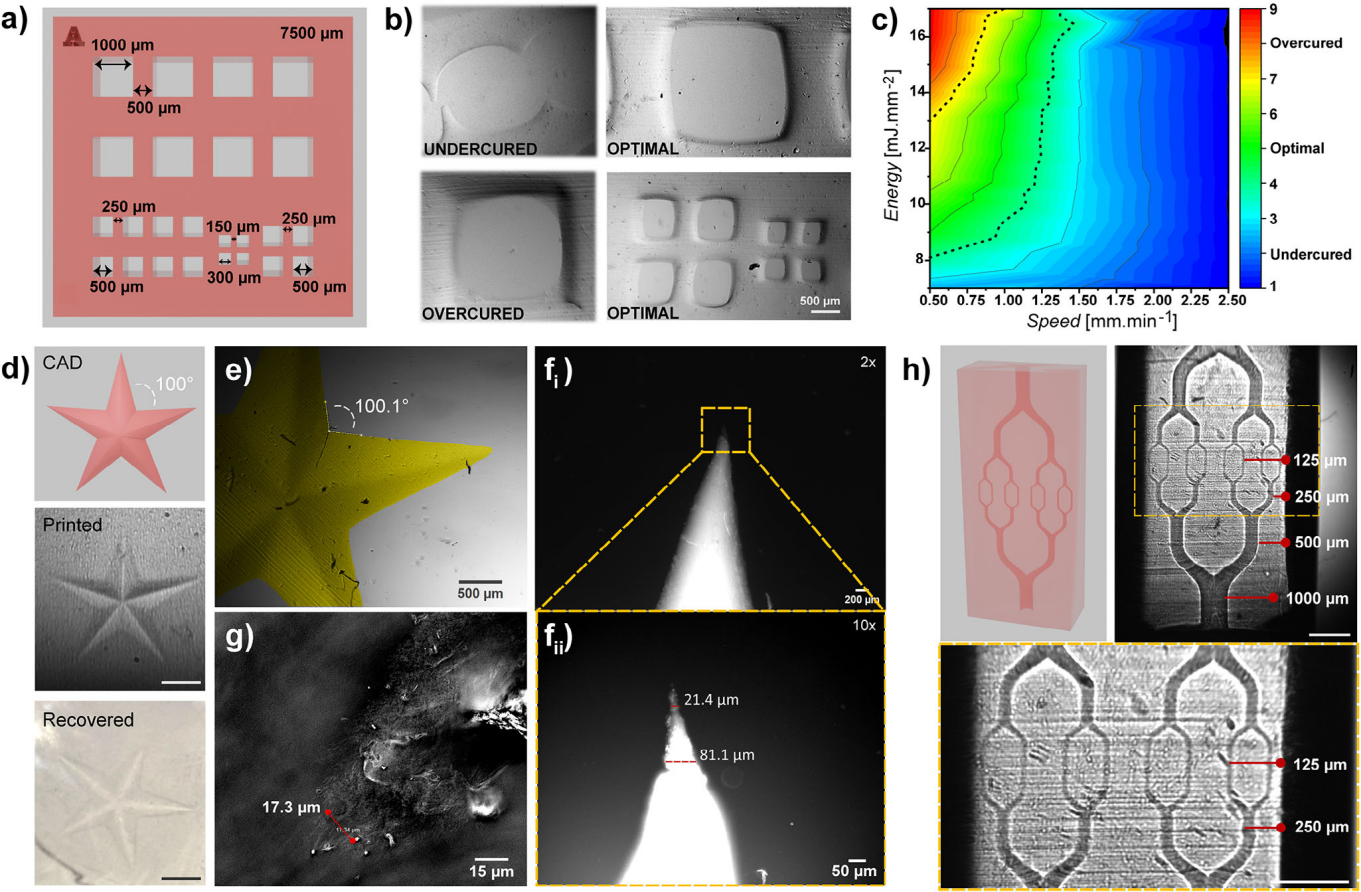
Parametric optimization for Xolographic 3D printing of GelMA‐based hydrogels. a) Print‐standards containing diverse‐sized positive and negative features, which were printed to evaluate the effect of different UV energy doses and printing speed on printing fidelity. b) Bright‐field micrographs of undercured, overcured, and optimal prints, obtained under different printing parameters. c) Heatmap visualizing the printing window in which parameters are optimized for an accurate print. d) 3D‐printed star using optimal printing conditions. Scale bars indicate 0.2 mm. e) Bright‐field micrographs of the printed star (digitally colored for visualization); f) fluorescence micrographs of a stained star (eosin Y) at 2x i) and 10x ii) magnifications; g) label‐free holotomographic image of a star tip. h) CAD‐model and a Schlieren photograph of a 3D printed capillary‐bed‐like structure showcasing negative features ranging from 1000 µm down to 125 µm. The striations along the UV beam direction within the light sheet are caused by an optical phenomenon similar to the principle of filament light biofabrication.^[^ ^23^ ^]^ Scale bars indicate 2 mm unless otherwise stated.

Achieving high‐resolution and high‐fidelity prints using Xolography requires a detailed understanding of the interplay between printing energy and speed. To explore these effects, we printed calibration standards containing a range of positive (1000 to 150 µm) and negative features (1000 to 300 µm) (**Figure** **4**a). GelMA‐based hydrogels were printed under varying UV irradiation energies and speed pairs, then recovered and analyzed via microscopy to assess print quality and dimensional fidelity. Specifically, Xolographic prints were quantitatively analyzed on the basis of their undercuring, optimal curing, or overcuring via their ratio of designed versus printed area (Figure 4b; Figure S5, Supporting Information). Systematic mapping of the effects of the print speed versus print energy yielded a parametric diagram that identified the high‐fidelity print window (Figure 4c). This revealed that Xolographic printing of GelMA‐based hydrogels could be achieved with speeds as high as 1.25 mm min^−1^ when working on 16 mJ mm^−2^. If lower energy doses are desired, then Xolographic printing of GelMA‐based hydrogels could be achieved at energy doses as low as 8 mJ mm^−2^ by lowering the print speed to 0.5 mm min^−1^ without losing print fidelity. This thus provides Xolographic printing of hydrogels with an operation window that balances speed and energy input to optimize printability and resolution for specific inks or geometrical print designs.

We next set out to determine the minimal achievable resolutions of Xolographically printed hydrogels. Five‐pointed stars spanning a width of 0.8 cm were printed within a minute to assess the minimal positive resolution (Figure 4d). Following recovery, microscopical analysis revealed excellent shape fidelity, with sharply defined edges across all three spatial dimensions and precise preservation of angles between the star arms (e.g., 100° design angles with 100.1° print angles) (Figure 4e). Fully swollen stars were labeled with eosin Y and analyzed on tip size using fluorescence microscopy, which revealed a minimal print of ≈20 µm (Figure 4f). Moreover, this observation was corroborated in a label‐free manner using holotomography, which is based on 3D refractive index tomograms of volume holographic gratings (Figure 4g).

In addition to a positive resolution, the negative resolution is of key importance to biofabricating multi‐scale hierarchical architectures such as those of native living tissues. Specifically, endowing engineered living tissues with vascularization is essential for its survival as it enables the gas and nutrient exchange that is necessary to support its metabolism. To this end, we investigated the minimal negative resolution of Xolographic printing of GelMA‐based hydrogels. Bifurcating vascular‐like structures with channel diameters ranging from 1000 to 125 µm were designed (Figure 4h). Schlieren imaging of Xolographic hydrogel prints revealed clearly defined channels for all tested sizes. This demonstrated that negative features as small as (at least) 125 µm could be Xolographically printed at high fidelity. Conclusively, when mediated by DPI, Xolographic printing of hydrogels attains the high resolutions necessary for tissue engineering and other soft material applications such as soft robotics or wearable sensors.^[^ ^21^, ^22^ ^]^

### 2.4 High‐Resolution Xolographic Printing of Complex‐Shaped Hydrogel Constructs

**Figure 5:**
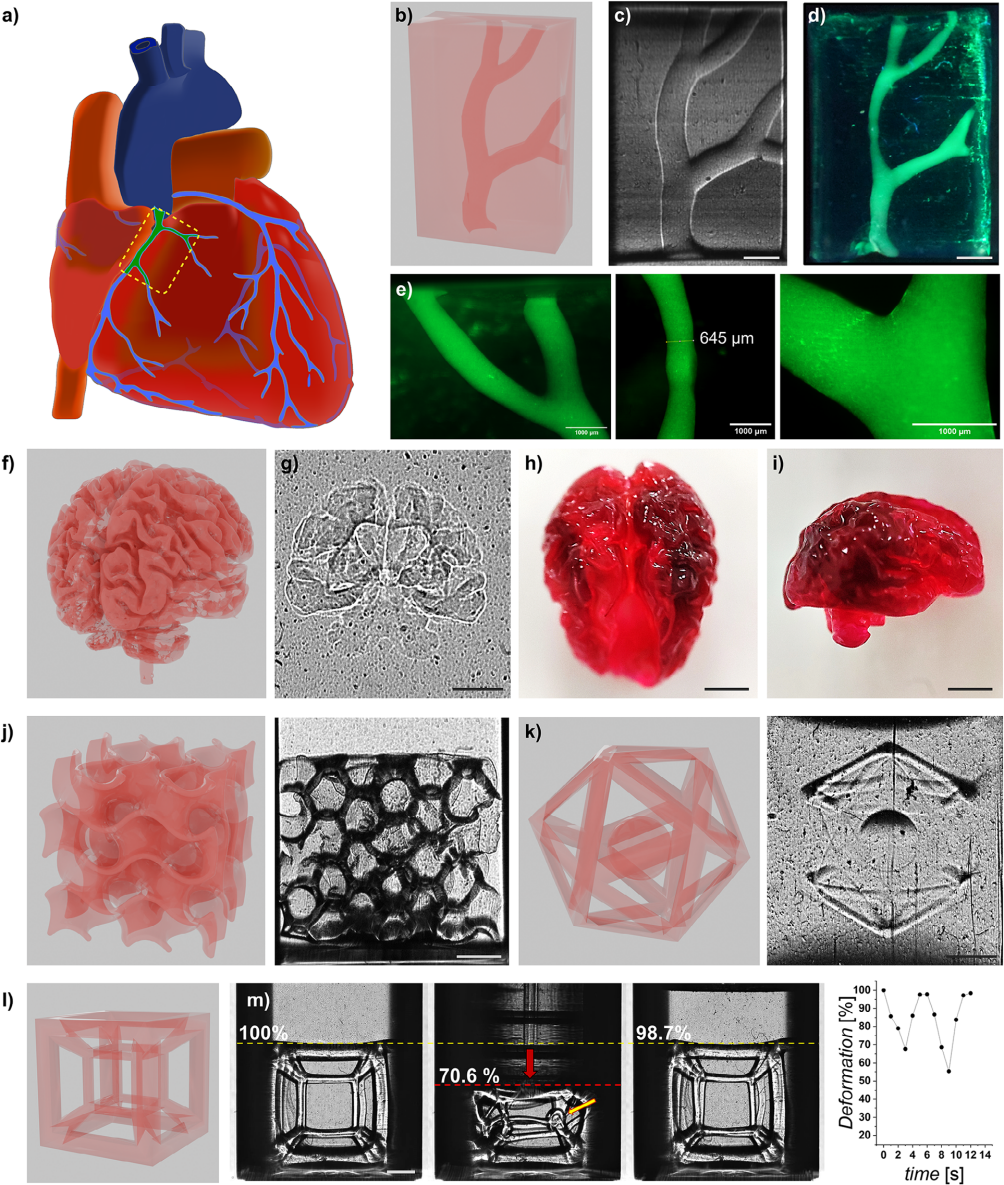
Xolographic printing of robust yet intricately structured hydrogels at high resolution. a) Schematic depiction, b) CAD model, and c) printed structure of a human heart highlighting a portion of the right coronary artery, which was based on a computed tomography image. d) The printed artery was perfused with fluorescent µ‐beads to e) visualize the negative space. f) CAD model and g) Schlieren photograph of Xolographic print structure of an MRI‐derived hypertrophic brain.^[^ ^25^ ^]^ h) Bottom‐view and i) top‐view photographs of the printed structure stained with eosin Y. CAD models and Schlieren photographs of printed structures of j) a gyroid,^[^ ^26^ ^]^ k) a free‐floating ball‐in‐an‐icosahedron,^[^ ^27^ ^]^ and l) a 3D projection of a tesseract.^[^ ^28^ ^]^ m) Schlieren photographs and deformation quantification of tesseract before, during, and after mechanical deformation. The yellow arrow highlights the presence of a flexural loop under deformation, which is then fully recovered, accenting the flexibility and robustness of the fine‐printed structure. Scale bars indicate 2 mm unless stated otherwise.

We next explored the capability of DPI‐mediated Xolography for printing complex shapes. To this end, we printed a CT‐derived 3D model of the right coronary artery of a human heart (**Figure** **5**a,b).^[^ ^24^ ^]^ The hollow and continuous nature of printed channels was visualized with Schlieren imaging (Figure 5c). Upon retrieval and allowing the prints to achieve their fully swollen status, we introduced fluorescent microbeads into the artery channels to confirm their perfusability (Figure 5d). Interestingly, this also revealed that swelling decreased the channel diameter, which could be leveraged as a predictable 4D print strategy to overcome minimal negative print resolution limits to allow for the fabrication of even perfusable channels with even smaller diameters. Moreover, the high print fidelity of these channels could be further observed by the accurate emulation in the natural slight variation in channel diameters that were encoded in the original CAD model (Figure 5e).^[^ ^24a^ ^]^ Regardless, Xolography's ability to create intricate vascularized tissue constructs could aid the creation of physiologically relevant and larger engineered tissues.

To further explore the fabrication of intricate 3D architectures featuring both finely resolved positive and negative structures, we printed an MRI‐derived model of a hypertrophic brain (Figure 5f). The undulating interwoven surfaces of the sulci and gyri in the brain's cortex brain present a unique challenge owing to its high‐resolution high‐curvature topography. These features were successfully Xolographically printed and maintained after recovery (Figure 5g–i). The ability to fabricate a delicate balance between positive and negative features was further corroborated by Xolographic printing of gyroid lattices. These lattices showcase the print precision in rendering interconnected closed and open geometries (Figure 5j; Video S1, Supplementary Video). In addition, we confirmed that this approach can be expanded to even more complex prints such as those including free‐floating objects such as a ball within an icosahedron (Figure 5k; Video S2, Supplementary Video). Notably, free‐floating objects are near‐impossible to print via 3D printing methods other than volumetric or embedded 3D printing. To demonstrate that the Xolographic prints possessed sufficiently high mechanical properties to allow for down‐stream handling, we printed a tesseract‐like structure featuring numerous overhangs, very thin struts, and complex intersections (Figure 5l; Video S3, Supplementary Video). Post‐recovery, the tesseract prints were able to withstand induced mechanical deformation allowing for physical compression of up to 50% without detectable plastic deformation (Figure 5m). These findings affirm the capability of Xolographic printing to produce highly intricate, application‐specific hydrogel architectures that retain their integrity under mechanical stress, opening new pathways for soft material engineering.

### 2.5 Xolographic Printing of Living Matter

**Figure 6:**
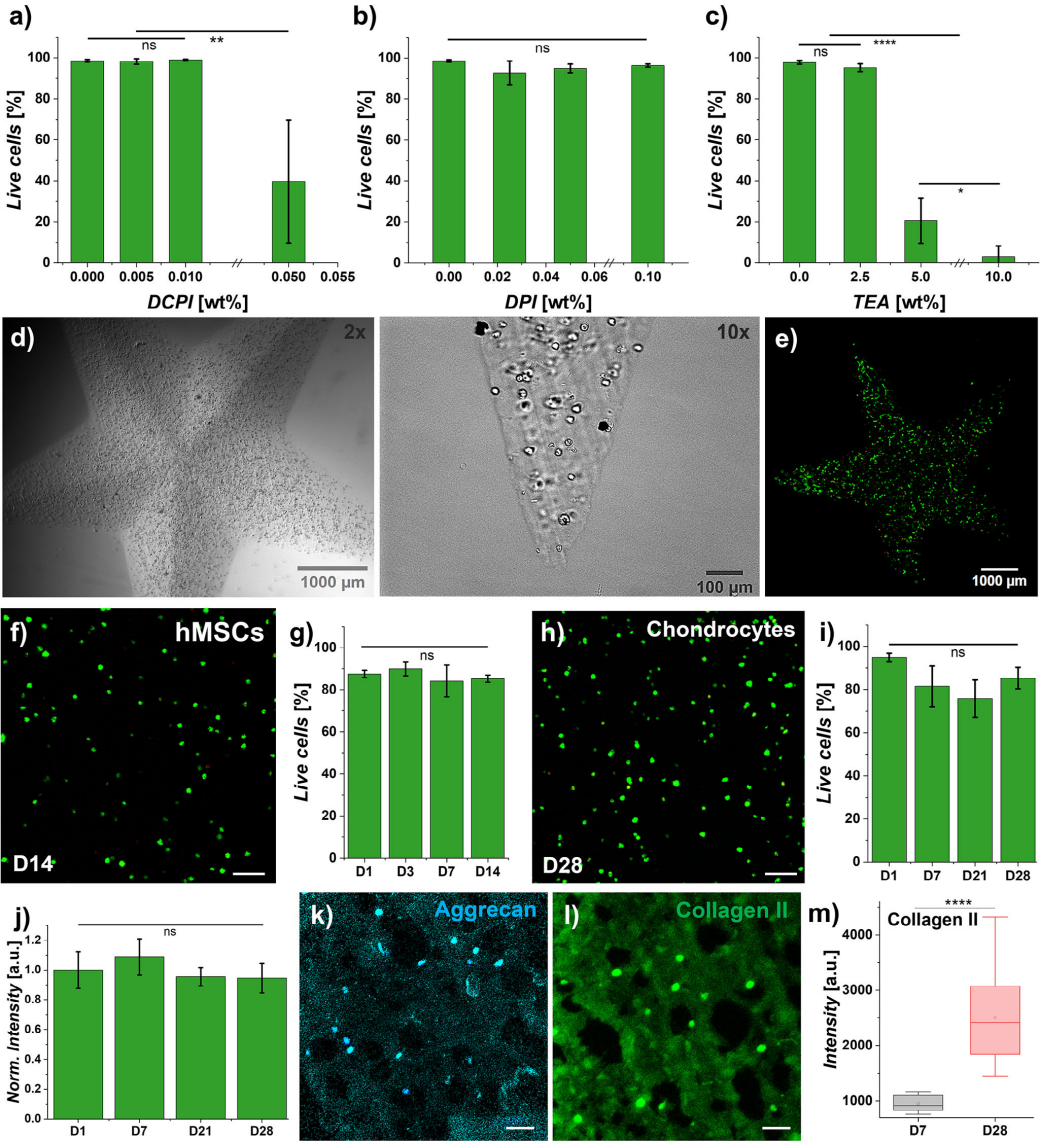
Bioxolographic printing of GelMA‐based hydrogels containing hMSCs is compatible with high cell viability, long‐term culture, and deposition of nascent proteins. Cytotoxicity of a) DCPI, b) DPI, and c) TEA. d) Bright‐field and e) fluorescence confocal micrographs (live/dead) of Xolographically 3D bioprinted cell‐laden stars two days after printing (hMSCs, 1 × 10^6^ cells mL^−1^). f) Live/dead and g) cell viability quantification of 3D‐bioprinted hMSCs and h, i) chondrocytes. Scale bars indicate  100 µm. j) Mitochondrial activity of chondrocytes that were Xolographically printed. Fluorescence micrographs of Xolographically printed chondrocytes stained for aggrecan k) and collagen II l) after 28 days of culture in a chondrogenic medium. Scale bars indicate 50 µm. m) Semi‐quantitation of differences in nascent COL2 deposition between day 7 and day 28. Mean ± SD shown, statistical analysis performed by one‐way ANOVA, Tukey's post‐hoc test, ***p < 0.001. The data shown is representative of three biological samples each one measured in triplicate.

We next determined whether Xolographic printing was compatible with the printing of engineered living materials, which is a process we termed Bioxolography. We, therefore investigated the cytocompatibility of the DCPI, TEA, and DPI as they are the fundamental components of Xolography. NVP was not included as a condition as it has already repeatedly been demonstrated to be cytocompatible at concentrations several folds higher than used in the here presented bio‐ink formulation.^[^ ^12^, ^29^ ^]^ It was revealed that DCPI exhibited no significant cytotoxicity at concentrations required for hydrogel 3D‐printing (0.005 wt.%), supporting cell viability >98% (**Figure** **6**a). Similarly, DPI showed no significant cytotoxicity at printing concentrations (Figure 6b). In contrast, the co‐initiator TEA demonstrated relevant concentration‐dependent cytotoxicity (Figure 6c). Although the origin of TEA cytotoxicity is currently unknown, we observed that TEA, as an organic base (pK_a_ = 7.74), can significantly alter the pH of PBS buffer when in high concentrations. Adjusting TEA's pH to a physiological level (7.2) neutralized its cytotoxic effects but failed to promote free‐radical photopolymerization (Figure S6, Supporting Information). To overcome this challenge and allow for Xolographic printing of living matter, we screened the effect of TEA concentration on cell survival and revealed that TEA concentrations up to 2.5 wt.% were cytocompatible (Figure 6c).^[^ ^12a^ ^]^ Indeed, formulating Xolographic print baths containing all optimized components (0.005 wt.% DCPI, 1000 mм DPI, 0.3 wt.% NVP, and 2.5 wt.% TEA) proved to enable rapid high‐resolution Xolographic printing of GelMA‐based hydrogels with excellent levels of cell survival (Figure 6d,e). While these prints maintained high resolution at 1 × 10^6^ cells mL^−1^, achieving higher cell densities will require precise optimization of bioresin optical properties to minimize cell‐induced light scattering during printing.^[^ ^30^ ^]^ Additionally, printing cm^3^‐sized constructs with high cell densities might impose metabolic constraints. To address this, functional vasculature bioprinting and/or bioresin supplementation with oxygen and nutrients might be required to ensure construct survival during extended printing and post‐implantation.^[^ ^31^ ^]^

The GelMA‐DPI‐NVP hydrogel solution was demonstrated to be compatible with volumetric printing of various cell types, including human mesenchymal stem cells (hMSCs) and primary chondrocytes. Specifically, hMSCs‐containing Xolographic prints were cultured in a growth medium for up to 14 days, showing excellent (>80%) cell viability (Figure 6f,g). Similar levels of cell viability were observed for Xolographically printed human primary chondrocytes (hPCs) cultured in a chondrogenic medium for up to 28 days (Figure 6h,i). To demonstrate cellular performance over time, we confirmed that the chondrocytes maintained their mitochondrial activity, indicating robust cellular health and continued metabolic activity (Figure 6j). Furthermore, the chondrogenic behavior of the printed cells was demonstrated using immunostaining for key biomarkers of cartilage. Specifically, the main components of cartilage tissue (e.g., aggrecan and collagen II) steadily increased over time (Figure 6k–m). This demonstrates that the novel three‐component formulation based on DPI offers the first solution that enables the Xolographic printing of living matter from polymer solutions at high resolution while preserving cellular functionality including the survival of cells as well as their ability to form *de novo* tissue via the deposition of nascent extracellular matrix proteins.

### 2.6 Xolography Facilitates Volumetric Light‐Dose Patterning of Mechanical Properties and Molecular Patterns

**Figure 7:**
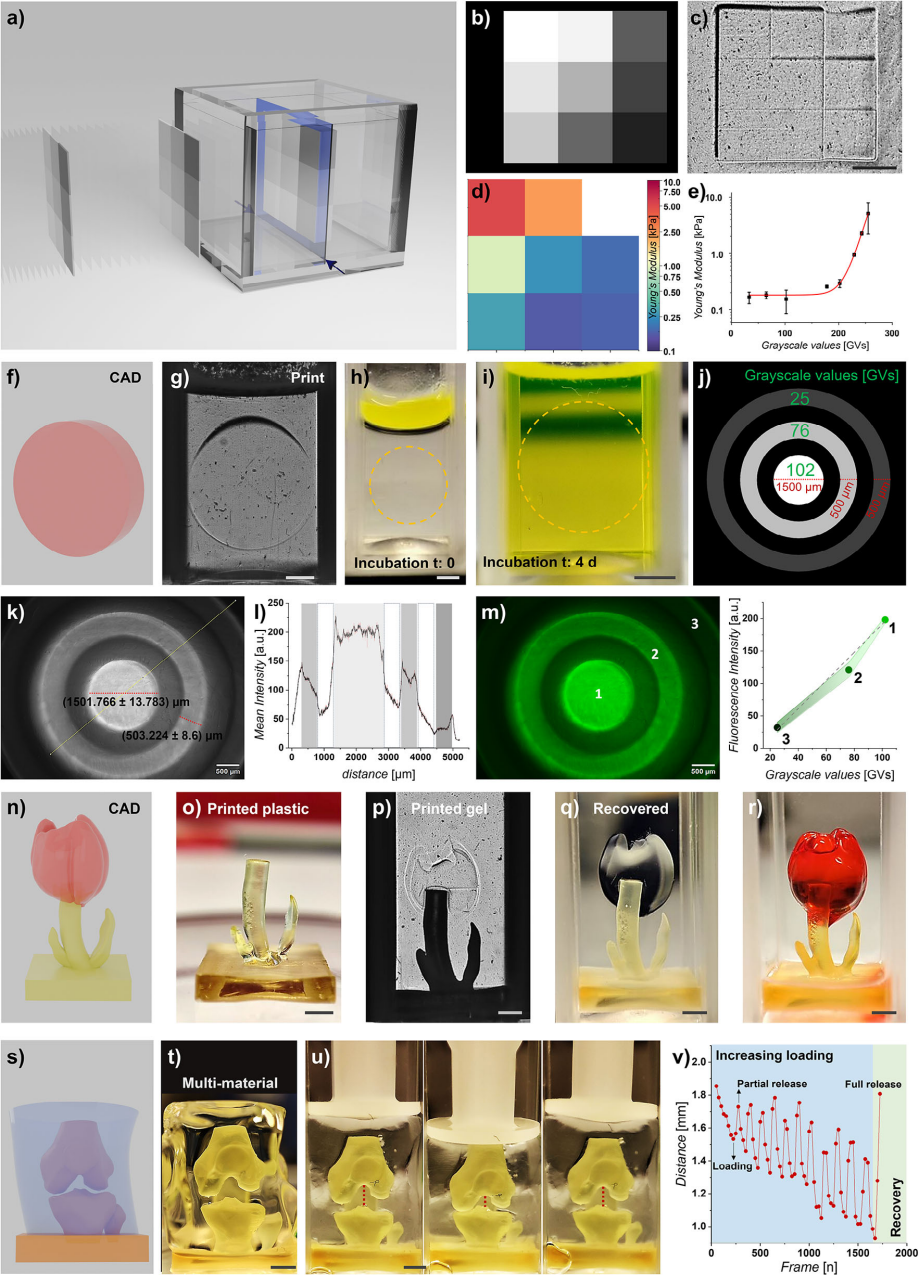
Anisotropic spatial patterning of light‐dose controlled mechanical and molecular properties, and multi‐material printing enabled by Xolography. a) Schematic depiction of volumetric light‐dose patterning based on grayscale video projections during Xolographic printing. b) Grayscale‐controlled projections dictate the light dose to c) spatially encode objects during fabrication. d) This allows for light‐dose‐dependent spatial control over the printed hydrogels' local elastic moduli, e) which intimately correlates with the projected gray values. f) CAD model and g) Schlieren photograph of a 3D‐printed GelMA hydrogel disc, which h, i) was post‐print soaked with a fluorescein dimethacrylate solution. Spatially controlled molecular patterns were encoded into the hydrogel disc via covalently bonding with the fluorophores via j) grayscale‐controlled projections. k) Fluorescence confocal micrography and l) line‐image‐based quantification revealed high‐resolution patterns, which m) of which the concentration was intimately grayscale‐dependent. Multi‐material 3D printing was demonstrated using a n) CAD model of a tulip of which o) a hard UDMA‐based resin stem was printed, p) which was overprinted with GelMA‐based petals as visualized using Schlieren photography. Photographs of multi‐material prints q) after recovery and r) after being stained with eosin Y. s) CAD model of a human knee and t) photograph of recovered multimaterial print in which hard bone structures were printed with UDMA and soft tissue with GelMA hydrogel. u) Photographs and image‐based quantification of inter‐osseous distance upon cyclical compression with progressively increasing mechanical loadings followed by full release of compression. Scale bars indicate 2 mm unless stated otherwise.

In comparison to other volumetric bioprinting techniques, Xolography offers the unique advantage of allowing for voxelated light‐dose‐based patterning of 3D printed constructs. Xolography's linear‐dose nature enables precise light‐dose spatial control of polymerization without the need for convoluted feedback mechanisms in contrast to its demand in computed axial lithography.^[^ ^5^, ^7^ ^]^ We anticipate that this inherent capability of Xolography can endow 3D bioprints with tailored mechanical properties or bioactive signaling gradients, thereby enabling the precise replication of natural tissue architectures. To demonstrate this feat, we exposed GelMA‐DPI‐NVP solutions to grayscale video projections to yield locally distinct amounts of photopolymerization (**Figure** **7**a,b). The projected light‐intensity pattern was visible using Schlieren imaging, suggesting local differences in crosslink density and consequent differences in light refraction (Figure 7c). Spatially resolved mechanical analysis using optical interferometry‐based nanoindentation confirmed spatial variations in elastic moduli in a manner that followed the projection pattern (Figure 7d). Indeed, the mechanical properties correlated directly with the grayscale intensity of the projection, confirming Xolography's ability for volumetrically controlled visible light‐dose patterning (Figure 7e). In a single step, with a single material, Xolography can create structures with a range of material properties. It is anticipated that this feat can be used to engineer living tissues with spatially resolved mechanical properties, which could aid in achieving locally controlled mechano‐induced programming of cell fate.

We next hypothesized that volumetric light‐dose patterning could also be leveraged to molecularly pattern biochemical information into Xolographic prints by spatially controlling the photochemical ligation of biomolecules onto hydrogels. To demonstrate this feat, fluorescein dimethacrylate was diffused into a Xolographically pre‐printed GelMA hydrogel (Figure 7f–i), which was Xolography overprinted using a multi‐light‐dosed grayscale pattern with a concentric ring‐like design (Figure 7j). After washing away unreacted fluorophores, brightfield and fluorescence microscopy revealed high‐resolution patterns that intimately followed the projected concentric ring design, which featured sharp delineations between patterned and nonpatterned regions (Figure 7k,l). Importantly, the fluorescence intensity was directly proportional to grayscale values, which confirmed light‐dose‐dependent molecular patterning (Figure 7m). This facile yet versatile approach highlights the potential of Xolographic printing to endow hydrogel and living matter with complex spatial patterns of molecular and biochemical cues, which possess the potential to orchestrate cellular behavior in a spatially controlled manner.

### 2.7 Xolography Facilitates Multi‐Material Printing to Create Complex Constructs

Among the key challenges faced by volumetric 3D‐printing technologies is their currently limited flexibility for multi‐material printing, which is a critical requirement to replicate the inherent anisotropy of living tissues. To address this, we combined Xolographic printing of hydrophobic and mechanically strong plastics with hydrophilic and mechanically soft hydrogels, which enabled the fabrication of complex constructs with drastically different mechanical and chemical properties within a single workflow. As a proof of concept, a tulip structure with rigid stems and soft petals was designed (Figure 7n). To realize this structure, the mechanically stiff stem was Xolographically printed using urethane dimethacrylate (UDMA), which was post‐print hardened using multi‐wavelength irradiation (Figure 7o). The stem was submerged in a GelMA‐based polymer solution, where the petals were Xolographically printed on top of the stem (Figure 7p). The resulting multi‐material tulip exhibited well‐defined and seamlessly integrated features that were sufficiently well‐integrated and mechanically stable to allow for recovery and subsequent handling steps such as being chemically stained (Figure 7q,r).

We fabricated a knee‐like structure to further demonstrate the potential of multi‐material Xolography for biomedical applications (Figure 7s). UDMA was used to fabricate a megapascal stiff bone‐shaped component, while GelMA hydrogel was overprinted to emulate the kilopascal soft surrounding tissues (Figure 7t). We next performed mechanical testing to empirically simulate top‐down compression to which a knee is subjected during physical activities, which was achieved by applying a compressive force that effectively compressed the soft material, which resulted in a reversible decrease in intra‐osseous space (Figure 7u). Moreover, increasing the progressively intense cyclical mechanical deformation of the Xolographically printed multi‐material joint construct allowed for up to 50% compression, which fully recovered its original shape upon release of the pressure (Figure 7v). This confirmed the effective integration of the two materials, with the structure maintaining elasticity and resilience under deformation and good shape recovery (Video S1, Supplementary Video). This feat can be of high value for soft robotics and tissue engineering, as tissue function relies on anisotropic mechanical properties to mediate both locally controlled mechanotransduction and organ articulation.^[^ ^22^ ^]^

## 3 Conclusion

This study demonstrates the versatility and potential of Bioxolography as a novel volumetric 3D‐printing technique for the rapid and high‐resolution fabrication of hydrogels and living matter with tailored mechanical and (bio)chemical properties. This was achieved by addressing the reactivity limitations of currently available water‐soluble DCPIs, by introducing DPI as a co‐initiator and NVP as a comonomer. This novel three‐part photochemical approach enabled the rapid 3D printing of cell‐laden GelMA solutions at <20 µm resolution with excellent cell viability. This supports the use of Xolography as an innovative enabling tool for engineering living tissues that could advance the creation of drug screening models, engineered meat, and organ replacements. Moreover, a key advantage of the linear photopolymerization nature of Xolography is that it offers unprecedented capabilities for volumetric light‐dose patterning. We demonstrated that this can be leveraged to locally modulate the stiffness within constructs as well as allow for spatially controlled molecular patterning of photoreactive compounds at high resolution. This reflects Xolography's potential for creating biomimetic architectures that replicate the mechanical anisotropy and biochemical gradients that exist in natural tissues. Furthermore, we demonstrated that Xolography is compatible with multi‐material printing to allow for the seamless and layerless integration of hard and soft materials to enable the fabrication of mechanically and chemically complex constructs, exemplified by the creation of a tulip structure and a knee‐like model. These proof‐of‐concept demonstrations highlight Xolography's unique ability to print structures that combine distinct mechanical and chemical properties within a single workflow. In conclusion, Xolography offers a robust and promising technologically‐advanced additive manufacturing platform that overcomes key limitations in volumetric 3D printing. By leveraging its inherent capabilities, Xolography could provide a pathway to fabricate constructs that could meet the mechanical, biochemical, and structural complexity of natural tissues, which positions (Bio)xolography as a novel key technology to advance other areas of regenerative medicine, soft robotics, and tissue engineering.

## 4 Experimental Section

### Materials

Endotoxin purified gelatin methacryloyl (GelMA; 86% degree of functionalization, 167 kDa; Rousselot, X‐Pure) was dissolved in phosphate‐buffered saline (PBS) and used at a final concentration of 10 wt.% unless otherwise specified. The dual‐color photoinitiator (DCPI 5000; xolo GmbH) was prepared as a 1 wt.% solution in triethanolamine (TEA; Sigma‐Aldrich) and mixed into GelMA solution at a final concentration of 0.005 wt.%. Subsequently, TEA was added to reach a final concentration of 2.5 wt.%. When described, the following comonomers or additives were introduced: poly(ethylene glycol) diacrylate (PEGDA; M_n_ = 575 g mol^−1^; Sigma‐Aldrich), 4‐arm polyethylene glycol acrylate (2 kDa; Creative PEGWorks), 8‐arm polyethylene glycol acrylate (10 kDa; Creative PEGWorks), *N*‐vinyl‐2‐pyrrolidinone (NVP; Sigma‐Aldrich), N‐vinylcaprolactam (NVC; Sigma‐Aldrich), and/or diphenyliodonium chloride (DPI; Sigma‐Aldrich). Urethane dimethacrylate‐based resin (UDMA‐based resin, xoloZero; xolo GmbH) was used as received, without further modification.

### Optical Characterization

Ultraviolet‐visible (UV–vis) spectroscopy measurements were performed using a Varian Cary 50 spectrophotometer. Samples were prepared in fluorescence cuvettes (BRAND GmbH & Co. KG, inner path length: 10 mm) and equilibrated at the required temperature for 20 min prior to measurement. Temperature control and stirring during measurements were integrated into the cuvette holder (Luma 4, Quantum Northwest, Inc.). The absorbance spectra of DCPI in its spiropyran state (absence of external irradiation) were recorded. To characterize light‐triggered isomerization to the merocyanine state, samples were irradiated perpendicularly to the measurement beam with a 375 nm LED (Thorlabs, M375L4, minimum output power 1270 mW at 1400 mA). The LED irradiation was collimated using an adjustable collimation adapter with an AR‐coated lens (Thorlabs, SM2F32‐A, coating range: 350–700 nm). Constant output power was maintained using a T‐Cube LED driver (Thorlabs, LEDD1B, maximum drive current: 1200 mA). Absorbance at 560 nm, corresponding to the merocyanine peak, was monitored over time at a frequency of 10 Hz to evaluate the thermal decay after irradiation ceased. For aqueous solutions, stirring was maintained at 1200 rpm during irradiation. In GelMA‐based formulations, stirring was omitted due to the high viscosity of the medium.

### Multi‐Wavelength Photorheology

The rheological properties of light‐irradiated ink formulations were measured on a Discovery HR20 rheometer (TA Instruments, USA) using 12 mm parallel plates with a bottom quartz plate. Samples were prepared as described for printing and equilibrated at 20 °C for 15 min prior to measurement. A 6‐wavelength high‐power LED source (Chrolis, Thorlabs) was employed to irradiate the samples during rheological testing. Time sweep measurements were conducted within the linear viscoelastic regime at a gap of 300 µm, controlled axial force (0.0 ± 0.1 N), frequency of 1 Hz, and strain of 1%. Light irradiation commenced 60 s after the onset of the time sweep, which provided simultaneous exposure to 365 nm (37 mW cm^−2^) and 565 nm (140 mW cm^−2^) light, which accounted for the dual‐wavelength absorption of the DCPI. At least three replicates were analyzed for each condition (n ≥ 3). All measurements were performed at 20 °C to mimic xolographic printing conditions. As gelatin‐based resins are a solid hydrogel at room temperature, traditional rheological sol‐gel transition analysis is challenged. Therefore, the hardening onset time and hardening speed of different resins were evaluated to compare their reactivity. Differences in these observables are likely caused by changes in crosslinking degree/polymer chain growth as well as the speed at which the reactions occur. The hardening onset time was defined as the point where the rheological curve deviated from its initial baseline behavior. This point was determined by mathematically fitting two intersecting lines at the inflection point. The hardening speed was calculated as the rate of modulus increase within the linear region following the hardening onset. To determine the linear viscoelastic regime, strain sweeps were performed across a range of 0.1% to 1000% strain at a constant frequency of 1 Hz, while frequency sweeps were carried out between 0.01 and 100 Hz at a fixed strain of 1%.

### Xolographic Printing Conditions

Following thorough mixing, all formulations were centrifuged (2500 rpm, 3 min) to remove gas bubbles and transferred into UV‐transparent cuvettes (Brand GmbH; transparency range: 230–900 nm) with dimensions of 10 × 10 × 35 mm. Computer‐aided design (CAD) models were sliced into 5 µm‐thick layers and converted into video format using xolid software (xolo GmbH). Xolographic printing was conducted using a xube printer (xolo GmbH). UV irradiation energy doses ranged from 1 to 20 mJ mm^−2^ and printing speeds ranged from 0.5 to 2.5 mm min^−1^, which were systematically screened and optimized for each (bio)ink formulation, and characterized as described below.

### Post‐Printing Processing

Recovered Xolographically printed hydrogels were washed repeatedly with phosphate‐buffered saline (PBS) at 37 °C to remove unreacted ink. No additional post‐printing hardening steps were applied unless otherwise stated. When post‐cured, samples were irradiated for 10 min with two simultaneous wavelengths (365 nm at 37 mW cm^−2^ and 565 nm at 110 mW cm^−2^). For fluorescent microscopy visualization or enhanced visibility (e.g., brain and tulip structures), samples were stained using an Eosin Y solution (0.5 wt.% in PBS). For hard plastic objects printed with UDMA‐based resins, post‐processing involved washing with isopropyl alcohol (IPA; Sigma‐Aldrich) and tripropylene glycol monomethyl ether (TPM; AprintaPro GmbH) to eliminate residual resin. The recovered printed objects were subsequently hardened by baking at 90 °C in an oven for 16 h, followed by multi‐wavelength irradiation at 365 nm and 565 nm for 20 min using the Chrolis multi‐wavelength setup (Thorlabs).

### Rheological Measurements

The rheological properties of printed and post‐cured constructs were measured using a Discovery HR20 rheometer (TA Instruments, USA) with an 8 mm parallel plate geometry. Samples were equilibrated at 20 °C before measurement. The linear viscoelastic regime was determined as previously described, and the storage modulus was recorded at 1 Hz and 1% strain for comparative analysis.

### Enzymatic Degradation

Collagenase IV‐mediated enzymatic degradation was performed following a previously reported protocol.^[^ ^32^ ^]^ Briefly, printed hydrogels were washed in PBS for three days to remove any residual photoinitiator or unreacted material. They were then incubated in collagenase IV solution (1.5 µg mL^−1^, 320 U mg^−1^) in PBS at 37 °C. Mass changes were recorded daily for seven days, with the collagenase solution refreshed every 24 h to maintain enzymatic activity. Degradation was assessed by calculating mass loss over time using Equation (1):

(1) $$Relative\;mass=m_{t}/m_{i}\;x\;100$$

where *m_t_* is the sample mass at a given time point, and *m_i_* is the initial mass.

### Post‐Print Characterization

Printability and print fidelity were evaluated using Schlieren photography and bright‐field microscopy, with a 3D‐printed standard serving as the benchmark. Microscopy images were analyzed with ImageJ software to measure the dimensions of printed and hollow areas (positive and negative features, respectively). Prints are classified as undercured when (Area_PRINT_ /Area_CAD_ > 1), overcured when (Area_PRINT_/Area_CAD_ < 1), or optimal when (Area_PRINT_/Area_CAD_ ≈ 1). The mechanical response of printed structures (e.g., tesseract and knee models) under dynamic deformation was evaluated (semi‐)quantitatively. Videos of the structures were recorded while applying external deformation at increasing strain. Frames were analyzed using ImageJ to quantify dimensional changes during deformation. For the tesseract model, the distance between the top and bottom sections was measured, while for the knee model, the distance between the femur and tibia was quantified.

### Cytotoxicity of Main Components

The cytotoxicity of DCPI, TEA, DPI, and PEGDA was assessed using 3T3 fibroblast monolayer cultures. Cells were seeded in 48 well plates (2.5 × 10^4^ cells/well) and incubated for 48 h. Following incubation, the culture medium was replaced with standard PBS containing varying concentrations of the tested compounds. For DCPI and DPI, DMSO was incorporated as solvent (0.5 wt.% in PBS). PBS and DMSO‐only conditions served as controls. Cells were maintained at room temperature for 30 min to simulate Xolographic printing conditions. PBS solutions were then removed, and a fresh culture medium was added. After 24 h, cell viability was assessed using a Live/Dead assay with calcein‐AM and ethidium homodimer. Fluorescence images were acquired using a fluorescence microscope (EVOS FL Imaging system microscope, ThermoFisher). The percentage of viable cells (green fluorescence) relative to the total cell count (green + red fluorescence) was determined using ImageJ software. For each condition, a minimum of 15 images from three independent replicates were analyzed. Data were reported as mean ± standard deviation (SD). Statistical differences between groups were evaluated using one‐way analysis of variance (ANOVA) followed by Tukey's post hoc test. A *p*‐value of <0.05 was considered statistically significant.

### Cell Isolation and Culture

Human mesenchymal Stem cells were isolated from patient samples as reported previously.^[^ ^33^ ^]^ Briefly, the ethical committee of the Medisch Spectrum Twente approved patient material usage, and written consent was obtained from all the samples (METC∖06003). The isolated bone marrow was used to collect all nucleated cells through aspirates, counted, and seeded in a cell culture flask at 5700 cells cm^−2^ in MSC growth medium that consists of aMEM (GIBCO) supplemented with FBS (10 v/v%), penicillin (100 mg mL^−1^), streptomycin (Gibco, 100 mg L^−1^), ascorbic acid (Sigma Aldrich, 0.2 × 10^−3^ M), Glutamax (Gibco, 1 v/v%), bFGF (Neuromics, 1 ug L^−1^) (added every time fresh) at 37 °C under 5% CO_2_. Cells were cultured with fresh medium refreshed every three days and passaged (five maximum) before harvesting using Trypsin‐EDTA (0.25 v/v%) at 37 °C. Human chondrocytes (hPCs) were isolated from the patient's tissues (nr 2020–7255) after approval by the local ethical committee in Radboud (CMS). Briefly, the cartilage tissues obtained from the non‐degenerated joints were digested using collagenase II in DMEM under agitation at 37 °C overnight. The hPCs recovered expanded in a growth medium consisting of DMEM high glucose supplemented with FBS (10 v/v%), Pen/Strep (Gibco, 1 v/v%), ASAP (Sigma Aldrich, 0.2 mм), L‐Proline (Sigma–Aldrich, 0.35 mм), and NEAA (ThermoFisher, 1x). The medium was refreshed twice a week before reaching ≈80% confluency, and then the cells were harvested using Trypsin‐EDTA (ThermoFisher, 0.25 v/v%). Cells were maintained in culture at 37 °C and 5% CO_2_.

### Bioxolographic Printing

GelMA(10 wt.%)‐DPI(1000 µм)‐NVP(0.3 wt.%) formulations were used for bioprinting. The formulation was prepared as described before, completing 2/3 of the final bioresin volume. Harvested cells were resuspended in PBS to make 1/3 of the final volume and pipette‐mixed within the reactive bioresin at 37 °C to make a final density of 1 × 10^6^ cells mL^−1^. The bioresin was transferred into UV‐transparent printing cuvettes and allowed to reach room temperature for 15 min. CAD‐designed projection such as squared sheets (e.g., 2 × 4 × 1 mm) were printed and immediately washed with PBS and 3 × cell culture media before transferring them to suspension well plates for long‐term culture. The printed structures with MSCs were cultured in proliferation medium (as mentioned above), and hPCs were cultured in a chondrogenic differentiation medium consisting of high glucose DMEM supplemented with Pen/Strep (1 v/v%), ASAP (0.2 mm), L‐Proline (0.35 mm), Sodium pyruvate (1×), ITS (1×), freshly added dexamethasone (1 × 10^−7^ m), and TGFβ‐3 (10 ng mL^−1^). The proliferation and differentiation medium were refreshed every three days, and the cell culture was maintained at 37 °C and 5% CO_2_. For the 3D‐bioprinting of more complex architectures (i.e., stars, tulips, or articular joints), iodixanol (24 wt.%) was added to minimize scattering events related to the refractive index difference between bioresin and cells.^[^ ^30^, ^34^ ^]^

### Cell Viability of Xolographically Printed Constructs

To evaluate cell viability, the printed structures at respective time points were incubated with calcein‐AM (indicating live cells, 2 µм) and EthD‐1 (dead cells, 4 × 10^−6^ м) (Thermo Fisher Scientific) for 30 min and visualized under 10× objective with confocal microscope (Zeiss LSM 880 airy scan). The fluorescence images were used to quantify cell viability as the live cells (calcein‐positive) ratio to the total number of cells. Mitochondrial activity was assessed using MitoTracker Green FM (Thermo Fisher Scientific, 5 µм). The printed structures were incubated with the MitoTracker solution for 60 min, followed by three PBS washes to remove the excess dye. Fluorescence imaging was performed using a confocal fluorescence microscope (Zeiss LSM 880 airy scan), and color intensity was quantified using ImageJ to analyze mitochondrial activity.

### Immunofluorescence Imaging

To evaluate chondrogenic differentiation of hPCs, the printed structures were cultured in a differentiation medium, fixed in formaldehyde solution (Sigma Aldrich, 4%) for 15 min, washed twice with PBS, and stored at 4 °C until further use. The printed structures were embedded in Cryomatrix (Thermo Scientific 6769006) cryo‐sectioned to obtain thin sections with a thickness of 20 µm. Before imaging, the sections were permeabilized with Triton X‐100 (Sigma Aldrich, 0.25 w/v%) in PBS for 30 min and washed twice with PBS. Blocking was performed using bovine serum albumin (BSA; Sigma Aldrich, 1%) in PBS for 60 min, followed by two PBS washes. Antigen retrieval for aggrecan was performed by incubating the sections in pepsin (0.1%) for 30 min at 37 °C. For immunostaining, the sections were incubated overnight at 4 °C with primary antibodies: anti‐collagen II (Abcam, ab34172, 1:100) and anti‐aggrecan (Abcam, ab3778, 1:100), prepared in BSA (1%). After primary antibody incubation, the sections were washed three times with PBS and subsequently incubated overnight at 4 °C with secondary antibodies: Alexa Fluor 488‐conjugated goat anti‐rabbit (Collagen II) and Alexa Fluor 647‐conjugated goat anti‐mouse antibodies (Aggrecan) (1:100 dilution). Nuclei were stained with DAPI (Thermo Scientific, 62248, 1:100) by incubating the sections at room temperature for 15 min, followed by washing with PBS. Fluorescence imaging was performed on the sections using a confocal microscope (Zeiss LSM 880 Airyscan) equipped with a 10x immersion objective. The acquired images were processed and analyzed using ImageJ.

### Grayscale Patterning

Grayscale patterning was performed using an ImageJ macro developed to process sliced STL models into grayscale patterns. Models were sliced into 5 µm‐thick layers using ChiTuBox software and exported as PNG images. The macro allowed user‐defined grayscale values (1–255) to be assigned to different sections of the object, offering precise control over the intensity of light projected during the printing process. The processed grayscale images were then combined into a video file and uploaded to the xube printer (xolo GmbH) for printing. This approach enabled spatial control over the amount of visible light deposited across the printing chamber.

### Stiffness Patterning

Squared sheets containing nine blocks with different grayscale values, ranging from 33 to 255), were Xolographically printed. Optical interferometry‐based nanoindentation (Pavone, Optics11 Life) was used to characterize the elastic modulus of each section and was correlated with projected grayscale values. Objects were indented to 4 µm using a 49 µm spherical probe with a cantilever stiffness of 0.490 N m^−1^. The resulting load versus displacement curves were fitted using a Hertzian contact model.

### Molecular Patterning

Hydrogel discs were incubated in a solution of fluorescein dimethacrylate (2.5 mg mL^−1^ in DMSO) at room temperature for four days to achieve uniform diffusion of the photoreactive dye throughout the hydrogel matrix. Following incubation, grayscale patterns were Xolographically projected onto the hydrogels, which covalently bonded the dye in a grayscale intensity‐dependent manner. To remove unreacted dye, hydrogels were washed extensively for two days. Fluorescence microscopy was used to analyze the resulting molecular patterns. Images were processed using ImageJ software to quantify the mean fluorescence intensity in each patterned region, corresponding to different grayscale values. A correlation between the projected grayscale intensity and the fluorescence signal was established (*n* = 5).

### Statistics

Data were expressed as mean ± standard deviation (SD). For each condition, a minimum of three independent experiments were performed. In all cases, a value of α < 0.05 was used for statistical significance. A one‐way ANOVA with a Tukey test of the variance was used to determine the statistical significance between groups.

## Conflict of Interest

The authors declare no conflict of interest.

## Supporting information



Supporting Information

Supplementary Video 1

Supplementary Video 2

Supplementary Video 3

## Data Availability

The data that support the findings of this study are available from the corresponding author upon reasonable request.
