# Supplementary material for: Bioxolography Using Diphenyliodonium Chloride and N‐Vinylpyrrolidone Enables Rapid High‐Resolution Volumetric 3D Printing of Spatially Encoded Living Matter
Source: Adv Mater. 2025 Apr 26;37(37):2501052. doi: 10.1002/adma.202501052 (PMC12447060; doi:10.1002/adma.202501052)
Supplement: Supplementary file 1 — Supporting Information [file ADMA-37-2501052-s004.docx]

Supporting Information

**Bioxolography using diphenyliodonium chloride and N-vinylpyrrolidone enables rapid high-resolution volumetric 3D printing of spatially encoded living matter**

Alexis Wolfel, Castro Johnbosco, Annalise Anspach, Marieke Meteling, Jos Olijve, Niklas Felix König, and Jeroen Leijten*


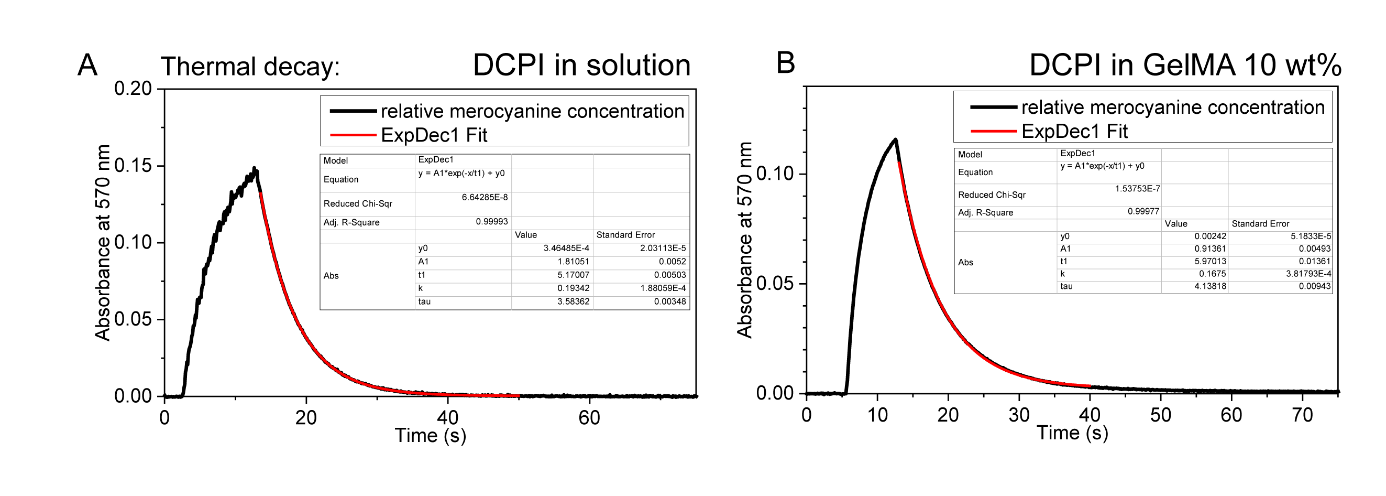


Figure S1. DCPI’s photoswitching kinetics at 25 °C showing generation of the merocyanine state during 375 nm UV irradiation (peaking), followed by thermal relaxation in the dark (decay). (A) DCPI in aqueous solution and (B) in GelMA 10 wt%.


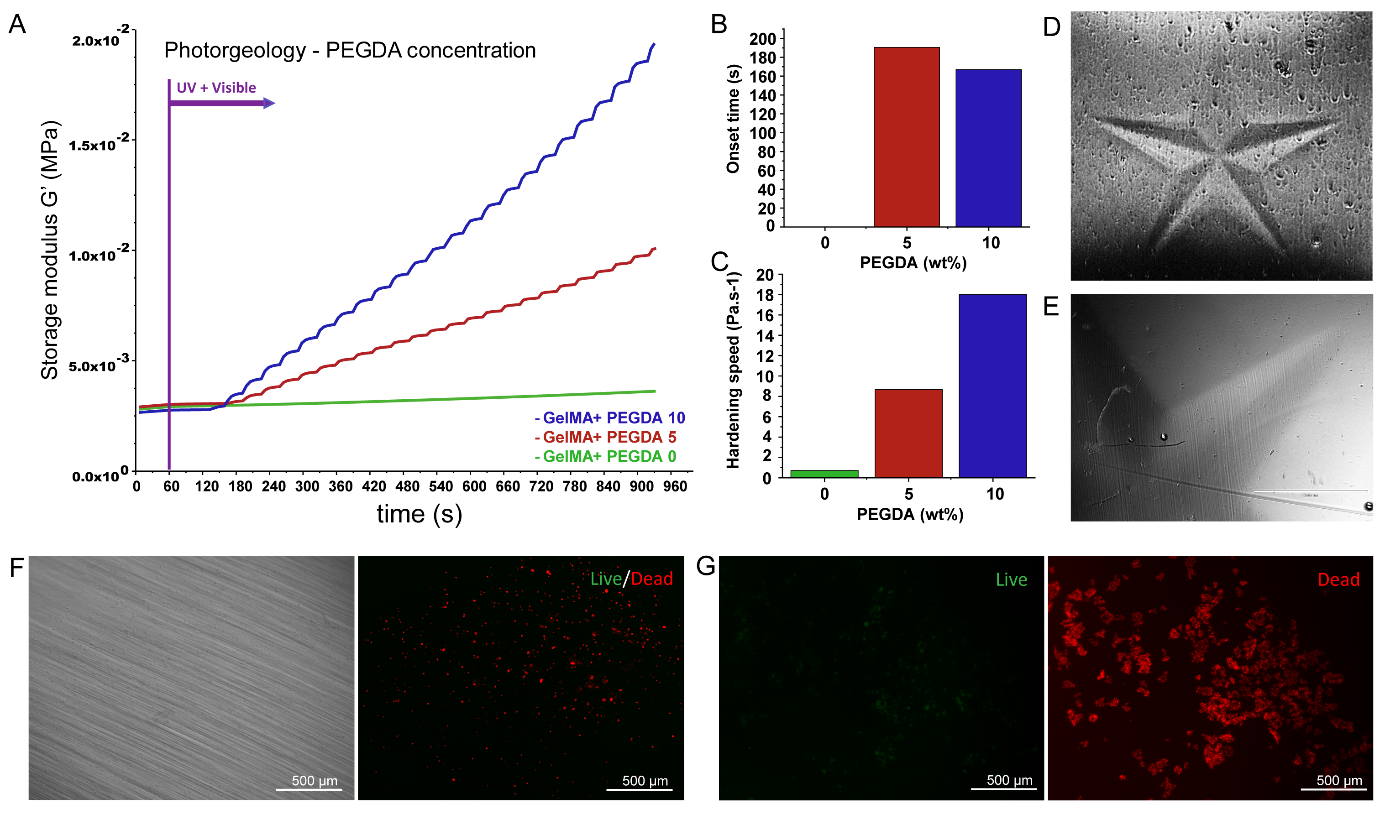


Figure S2. (A) Dual-color photorheology (λ = 365 nm + 656 nm) of GelMA formulations with increasing concentrations of PEGDA. (B) Effect of PEGDA in photopolymerization onset time and (C) hardening speed. (D-E) Xolographic printing of GelMA(10%)PEGDA(10%) formulation (top: schlieren imaging; bottom: BF-microscopy). (F) PEGDA brightfield and live/dead images of xolographically printed constructs using GelMA(10%)-PEGDA(10%) hydrogel formulation and hek293 cells. (G) Live/dead images of hek293 cells incubated with PEGDA 5 wt% for 30 min.


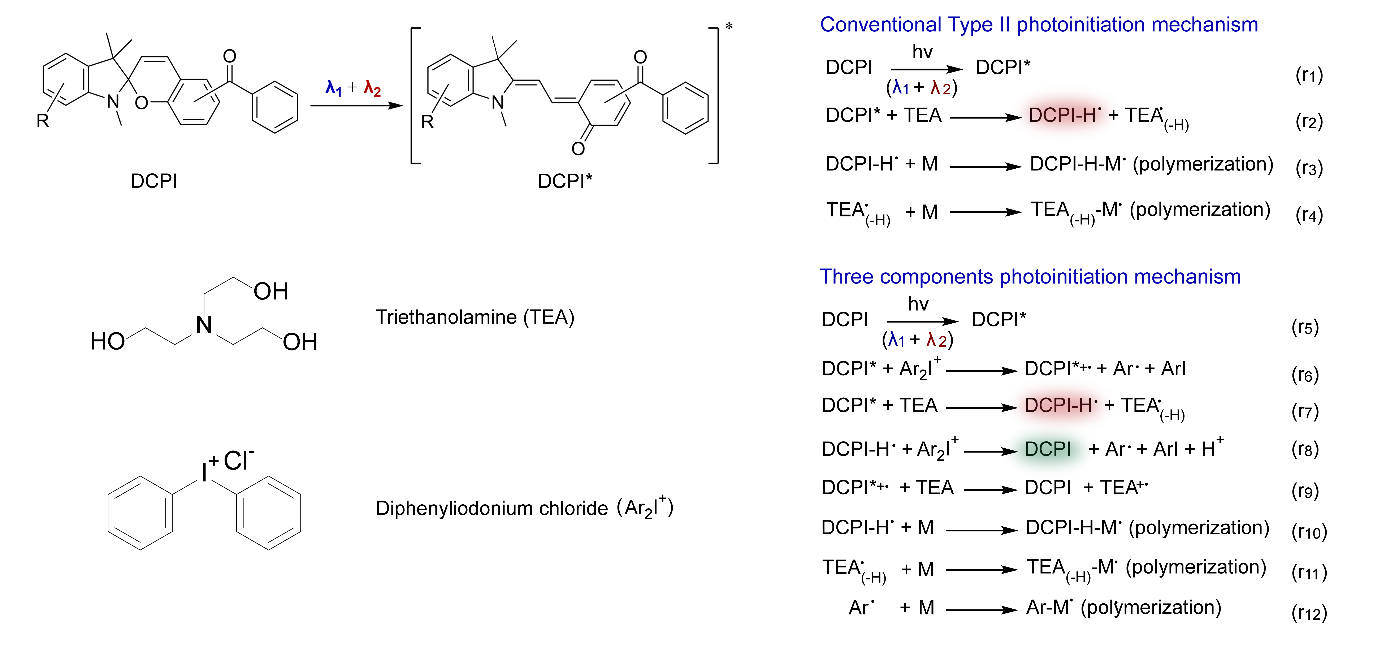
Figure S3. Proposed mechanism for the three-components photoinitiation system between DCPI, DPI and TEA. DCPI is a benzoyl-based type II photoinitiator (PI) which, in conventional photoinitiation systems, typically works by association with a tertiary amine such as TEA to promote free radical polymerizations.^[1]^ Upon light irradiation, a type II PI such as DCPI will be electronically excited (DCPI*) and react with TEA (an e^-^ donor), to form reactive radicals. DCPI and TEA radicals will then promote polymerization. However, the introduction of a iodonium salt (DPI, here depicted as Ar_2_I^+^ for clarity) has been previously proposed in combination with other benzoyl-based type II PI, and a tertiary amine, to form a three-component photoinitiation system with improve photopolymerization efficiency. Although it remains to be experimentally verified, we hypothesize that our three-component PI system will operate with a similar mechanism to that proved for other equivalent systems.^[2]^ Such systems have also demonstrated a dramatic increase in photopolymerization efficiency upon the addition of DPI, and proposed a **redox catalytic cycle** as a mechanism where the PI is regenerated during the reaction. This slows down PI consumption while speeds up the free radical polymerization. In our PI system, the radical formed from DCPI (DCPI*, red circle in r7) during reduction with TEA would be regenerated to its original state (DCPI, green circle in r8) by reaction with DPI, thus recovering its availability to promote new photopolymerization.


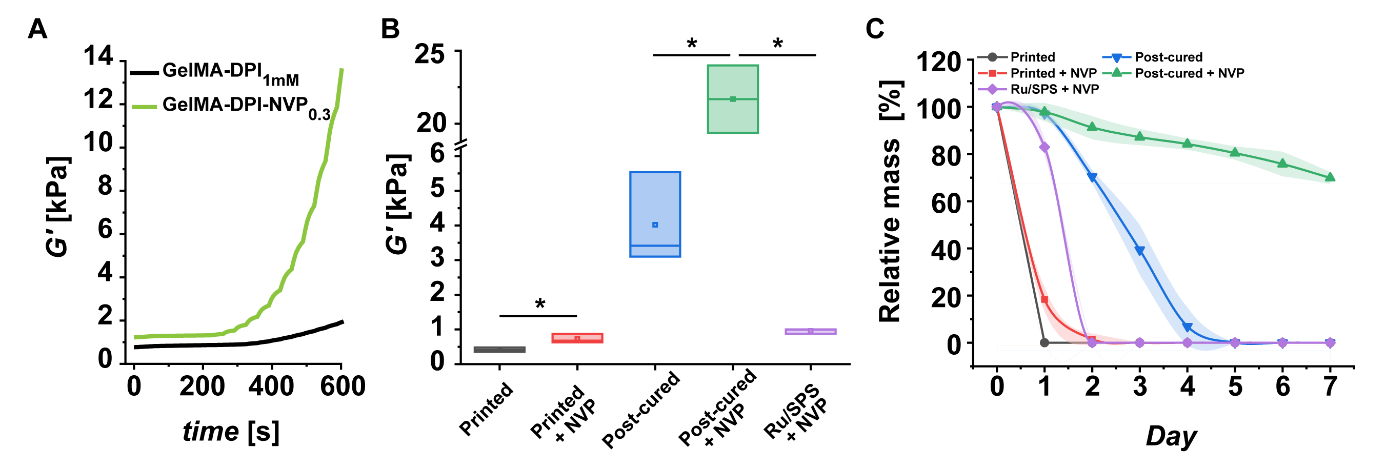


Figure S4. (A) Photo-rheology of GelMA_10%_-DPI_1mM_ and GelMA_10%_-DPI_1mM_ -NVP_0.3%_ showing the effect of NVP on photopolymerization onset time and hardening speed. (B) Influence of NVP 0.3% on the storage modulus (G') of xolographically printed and post-cured GelMA_5%_-DPI_1mM_ constructs. Hydrogels crosslinked using a standard photo-initiator (Ru/SPS) were prepared for comparison with DCPI-initiated gels (n = 3). (C) Collagenase IV-mediated enzymatic degradation of printed and post-cured constructs over time. (n = 3).


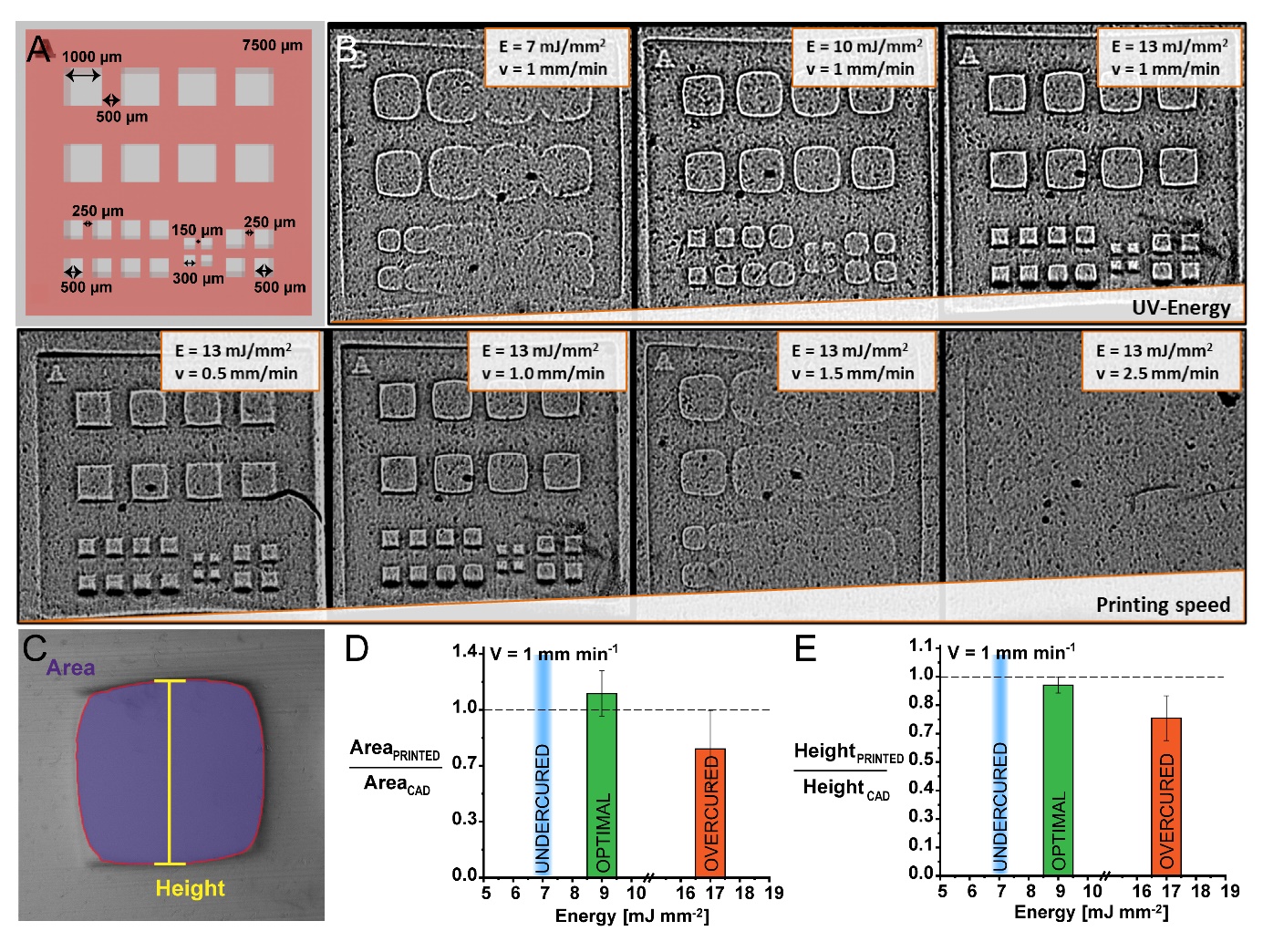


Figure S5. Evaluation of printing fidelity over GelMA-based printed hydrogels. (A) Designed standards for printing. (B) Schlieren images of printed structures under different printing conditions, before recovery from printing vat. (C) Microscopy image analysis over recovered prints: area and height of negative features (1000 µm width holes) was measured with ImageJ to compare them with the area (D) and height (E) intended in the CAD design.


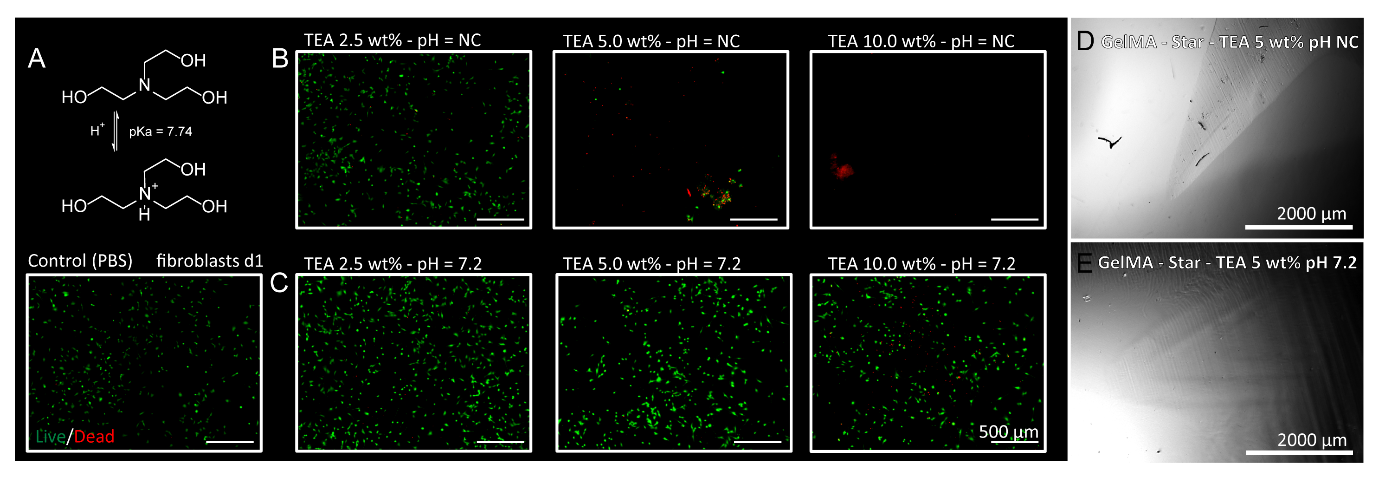
Figure S6. (A) Acid-base equilibrium for triethanolamine (TEA). (B) Cytotoxicity of TEA in solutions with not-controlled pH (pH = NC); and (C) in solutions with controlled pH (pH = 7.2). (D) Microscopy images of printed stars (arm) using (D) a TEA solution without pH control (final pH ~ 9) and with pH control (final pH = 7.2).

References

[1] A. Balcerak, J. Kabatc-Borcz, Z. Czech, M. Bartkowiak, *Polymers (Basel)* **2023**, 15.

[2] S. Liu, D. Brunel, G. Noirbent, A. Mau, H. Chen, F. Morlet-Savary, B. Graff, D. Gigmes, P. Xiao, F. Dumur, J. Lalevée, *Materials Chemistry Frontiers* **2021**, 5, 1982.
